# Systemic Inflammation: Methodological Approaches to Identification of the Common Pathological Process

**DOI:** 10.1371/journal.pone.0155138

**Published:** 2016-05-06

**Authors:** N. V. Zotova, V. A. Chereshnev, E. Yu. Gusev

**Affiliations:** 1 Laboratory of immunology of inflammation, Institute of immunology and physiology of UB RAS, Yekaterinburg, Russian Federation; 2 KCS “Experimental physiology and immunochemistry”, Auto Federal State Autonomous Educational Institution of Higher Professional Education “Ural Federal University named after the first President of Russia B. N. Yeltsin”, Yekaterinburg, Russian Federation; University of Leicester, UNITED KINGDOM

## Abstract

We defined Systemic inflammation (SI) as a “typical, multi-syndrome, phase-specific pathological process, developing from systemic damage and characterized by the total inflammatory reactivity of endotheliocytes, plasma and blood cell factors, connective tissue and, at the final stage, by microcirculatory disorders in vital organs and tissues.” The goal of the work: to determine methodological approaches and particular methodical solutions for the problem of identification of SI as a common pathological process. SI can be defined by the presence in plasma of systemic proinflammatory cell stress products—cytokines and other inflammatory mediators, and also by the complexity of other processes signs. We have developed 2 scales: 1) The Reactivity Level scale (RL)–from 0 to 5 points: 0-normal level; RL-5 confirms systemic nature of inflammatory mediator release, and RL- 2–4 defines different degrees of event probability. 2) The SI scale, considering additional criteria along with RL, addresses more integral criteria of SI: the presence of ≥ 5 points according to the SI scale proves the high probability of SI developing. To calculate the RL scale, concentrations of 4 cytokines (IL-6, IL-8, IL-10, TNF-α) and C-reactive protein in plasma were examined. Additional criteria of the SI scale were the following: D-dimers>500ng/ml, cortisol>1380 or <100nmol/l, troponin I≥0.2ng/ml and/or myoglobin≥800ng/ml. 422 patients were included in the study with different septic (n-207) and aseptic (n-215) pathologies. In 190 cases (of 422) there were signs of SI (lethality 38.4%, n-73). In only 5 of 78 cases, lethality was not confirmed by the presence of SI. SI was registered in 100% of cases with septic shock (n-31). There were not significant differences between AU-ROC of CR, SI scale and SOFA to predict death in patients with sepsis and trauma.

## Introduction

Today, criteria of systemic inflammatory response syndrome (SIRS) are the following (≥2 criteria from 4): 1) Body temperature ≥38C or ≤36 C; 2) Frequency of heartbeat rate > = 90; 3) Respiration rate >20 min or hyperventilation (PaCO_2_≤32 mm hl); 4) Blood leukocytes >12x10^9^/ml or <4x10^9^/l, or the number or immature forms > 10%; these criteria formalize the clinical diagnosing of sepsis if there is an inflammatory source [1]. It is clear that criteria of this syndrome do not directly reflect the key pathogenic phases, lying in the base of critical condition developing. The wide range of existing criteria for prognosis and estimating of critical complications can be structured in individual resuscitation syndrome, for example, multiorgan dysfunction syndrome (MODS) and disseminated intravascular coagulation (DIC) [2]. Development of these and other resuscitation syndromes is pathogenetically linked with the phenomena of the Systemic Inflammatory Response (SIR), which is usually associated with “Systemic Inflammation” (SI). However, from the position of common pathology there is a question we have to answer—can Systemic Inflammation be characterized with clinical interpretation of inflammatory processes, or should it be considered as a separate form of a common pathological process?

The content of classical inflammation is the response of the organism as a whole to local injury. Herewith, two non-identical levels of inflammation response can be marked out: local—from the inflammatory focus and systemic- SIR. Mechanisms of the first level are aggressive not only for injury factors but also for the organism’s tissues. SIR’s mechanisms are directed to support mechanisms of inflammatory focus and at the same time to intensify buffer systems, which prevent aggressive biofactors coming out from the inflammatory focus at the systemic blood flow. The mechanisms of SIR in classical variants of inflammation are the following: stress-reaction of neuroendocrine system, psychasthenia, high temperature, output of white blood cells to circulation from vessel and bone marrow depot, leucopoiesis strengthening, acute-phase response of the liver, generalized immune response manifestations, and some other mechanisms that should occur with the certain type of the common adaptive syndrome, connected with inflammation. It's easy to see that SIRS criteria primarily reflect manifestation of these mechanisms. The shift of classical inflammation to Systemic Inflammation is possible after mutation of the quality of inflammation program into another inflammation program with another content. In a few Russian publications [3–6] we tried to prove the necessity of SI consideration from the position of an independent type of the common pathological process and we have formulated the following definition: «Systemic inflammation—is a typical multi-syndrome, phase-specific pathological process, evolving at systemic injury and characterized by the total inflammatory reactivity of the endotheliocytes, plasma and blood cell factors, connective tissue, and, at the terminal stage—microcirculatory disorders in vital organs and tissues” [3]. Thereby, the fundamental difference between classical inflammation and SI is not the presence or absence of systemic changes, but changes in quality content, because of a shift to the system level of biologically aggressive mechanisms that were intended for local use inside the inflammatory focus. These include the following: systemic activation of endotheliocytes and vessel macrophages; proinflammatory transformation of structure and function of microvessels; microthrombosis in postcapillar venules; intravessel activation of complement and hemostasis; excitotoxicity—toxic accumulation of inflammatory mediators in blood; systemic degranulation of mastocytes, and some other mechanisms, intended for acting in the area of injury. The transformation of stress-reaction in the hypothalamic-pituitary-adrenal system to distress-reaction is specific for SI, but not attributive. Thereby, the principal distinguishing feature of SI appears to be a transfer of program mechanisms of inflammatory focus to the systemic level in response to systemic effect of injuring factors. Herewith, systemic injury phenomena development is connected not only with critical changes of some homeostasis parameters, but also with coming out of microbe and endogenic molecular patterns (for example products of tissue degradation) to the system blood stream, so called—danger-associated molecular patterns (DAMP) [5]. Acting power of injuring factors must overcome buffer barriers, which prevent systemic activation of proinflammatory mechanisms, genetically intended for use only in local inflammatory focus.

The initiation of proinflammatory cell-stress program is a common mechanism for activation of different injuring factors or threat of future damage, on this base complex, integral programs of different variants of cell-cooperations are forming, determining the development of one or another type of inflammatory process. Thus, the base of SI is a mosaic development of cell-stress on the systemic level that can be characterized as two alternative variants (stages) according to the effects of injuring factors [5]: 1) realization of resistance strategy—high proinflammatory activity, in prejudice of physiological functions; 2) realization of tolerance strategy—decease in cell activity, both physiological and proinflammatory. The dynamics of SI development is determined by the strength of the primary (initial) systemic damage, the development of secondary systemic damage phenomenon (self-developing process) and the ratio of two variants of cellular stress at the organism’s level [6]. Factors of the secondary systemic damage include: impairment of a number of homeostasis parameters associated with microcirculatory disorder development; entry into the bloodstream DAMP—products of tissue degradation and microbial endotoxins (translocation through mucosal barriers); cytotoxic effect of free radicals and proteases in the intravascular environment during the systemic activation of phagocytes [5].

In this case, we conventionally distinguish two versions of SI, depending on the intensity of system damage factors. The first option—a "breakthrough": for hyperacute, the primary focus of inflammation is absent or its role in the development process is a small, fast (during the day) change from a hyperresponsiveness phase to a more sustainable hypoergical (depressive) phase, the prognosis is generally unfavorable. Examples include fulminant sepsis (we have 1 case), amniotic fluid embolism (5 cases), lethal poisoning with acetic acid (6 cases), some options include injury and acute hemorrhage (11 cases, monitoring of the process in the first days of its development). Theoretically, this variant of SI, upon the dynamics of clinical manifestations and characteristics of the damaging factor, in addition, can include the following disorders: blood transfusion shock; crush syndrome; entry of biological poisons into the bloodstream that provoke intravascular hemolysis, activation of the complement system and hemostasis. The second option—"punching": the gradual transformation of the classical inflammation into the systemic; the presence of the transition zone, the essential role of the primary focus of inflammation; dynamics can be characterized by a sequence of several phases of hyperreactivity or gradual development of an immunosuppressive state; typical for sepsis and most types of injuries [6].

Individual manifestations of inflammation—the formation of cellular infiltrates of phagocytes and SIR (for example, the accumulation of inflammatory factors in the hemolymph) already observed in invertebrates (e.g. Nematoda, Annelida, Arthropoda, Mollusca, Echinodermata, Chordata) [4]. Systemic inflammation as a typical pathological process is possible in principle not only in humans, but also in other species of the class of mammals. Some of its manifestations may be detected in birds, while other classes of vertebrates, despite having had a greater or lesser degree of pro-inflammatory response of microvessels in the area of tissue damage, the development of SI as a holistic process is not obvious. The cause of this pattern is that, mammals have a more developed blood microcirculation and homeostatic system (e.g. they have a nuclear-free platelates) than other animals. A regulatory cooperation of main components of exudative-vascular complex (endothelium of postcapillaries, mast cells, homeostasis system and complement proteins) and a cytokine network in mammals are at a high level [4]. These differences between mammals and other animals result in mammals having some advantages when developing inflammation, e.g. the possibility of purulent inflammation. Although, such advantages can lead to the development of life threatening SI in case where damaging factors have systemic effect. One of the key objectives of the SI model description is the definition of methodological approaches for the evaluation of this common pathological process.

The purpose of the work: to determine methodological approaches and specific methodological solutions to the problem of identification of systemic inflammation as a typical pathological process.

## Materials and Methods

### Research of Systemic Inflammation: Methodology and Methods

The complexity of the process, a wide range of methods used to identify some of its manifestations, and theoretical and practical problems prejudge the usage of different methodological approaches. For example, one of the promising methods of lifetime assessment of microcirculatory disorders is the technology of side dark-field video microscopy (SDF microscope) and near-infrared spectroscopy (NIRS technology) [7].

Our approach boils down to two basic principles: 1) identification of systemic cellular stress according to accumulating cytokines and other inflammatory mediators (one of the manifestations of SIR) in the blood; 2) identification of other components of SI-process-complex, including in this case the following features: systemic alterations, organ dysfunction, systemic microthrombogenesis and distress reactions of the hypothalamic-pituitary-adrenal system [3, 6].

#### Identification of Systemic Inflammatory Response Intensity

Chaotic changes in SIR indicators in the blood and frequent inability to clearly differentiate the local production of cytokines from the systemic were taken into consideration. In order to solve this problem, the integral SIR rate was calculated on the base of detecting plasma C-reactive protein (CRP) and four cytokines: interleukins (IL)—IL-6, IL-8, IL-10 and tumor necrosis factor alpha (TNFα) in the form of a semi-quantitative Coefficient of Reactivity scale—CR (0–16 points), designed for statistical analysis of intergroup differences (patented method) [8]. For this purpose, for all five indicators, the serial numbers of ranges of concentrations in plasma with different pathogenetic and diagnostic significance were defined. The starting point for the formation of these intervals is the upper level of the rate for each factor, and the indicated ranges formed on the basis of the rate of excess top-level standards. Further, these ranges were fixed numerically as an individual reactivity index. The sum of the three largest values of the reactivity index of 5 factors used (2 of 5 smallest indices were excluded) determines the value of the CR in each case in Table 1.

**Table 1:** Calculation of reactivity index and coefficient of reactivity (CR).

Factor	UMN	Individual reactivity index (0–6) ranging according to the multiplicity of UMN-exceeding						
		0	1	2	3	4	5	6
IL-8	10 pg/ml	≤1	≤2,5	≤10	≤50	≤250	>250	no
IL-6	5 pg/ml	≤1	≤2	≤8	≤40	≤200	>200	no
TNFα	8 pg/ml	≤1	≤2	≤5	≤20	≤100	>100	no
IL-10	5 pg/ml	≤1	no	≤2	≤5	≤20	≤100	>100
CRP	10 pg/ml	≤1	≤3	≤15	>15	no	no	no

UMN—upper meaning of the norm in plasma. The sum of three maximal reactivity index values for each patient gives the value of the CR score (0 to 16). The use of other alternatives, criteria ranges of multiplicity exceeding UMN must be set individually, based on the characteristics of the particular factor and the method used, it’s registration in the blood plasma.

At the same time we did not differentiate the pro-inflammatory cytokines and conditionally anti-inflammatory cytokine IL-10, as their accumulation dynamics fundamentally are not divided into separate phases. According to the number of individual reactivity indices, the priority is of IL-10 (6), since its high concentration is mostly related with the most critical variants of SI. On the contrary, discrimination in this regard of CRP (used only 3 slots), is connected with the fact that it is a factor of an acute phase response that is not directly associated with the development of proinflammatory cellular stress and SI as a whole, but in some cases it may indirectly confirm the presence of SI.

To carry out the frequency analysis of SIR, the CR scale (0–16 points) was transformed into a more fundamental scale of reactivity levels (RL) from 0 to 5 points—RL (CR points): RL-0 (0–1), RL-1 (2–4), RL-2 (5–7), RL-3 (8–10), RL-4 (11–13), and RL-5 (14–16) [8]. At the same time the RL scale allows a qualitative assessment of the estimated SIR for a particular patient: RL-0—the level of physiological norm; RL-1—confirms SIR (in the classical inflammation), but excludes the development of SI; RL-2—is typical for classical inflammation, but it is also possible in some versions of the depressive phase of SI; RL-3—the zone of uncertainty; RL-4—typical for the hyperergic option of SI, the likelihood of developing classical inflammation is low; RL-5—confirms the presence of SI. Thus, the scale RL has a wide range of overlap for classical inflammation and SI.

#### Identification of Systemic Inflammation

Taking the information stated above into account to identify SI, its phases and structure complex processes, we have used an even more integral index—the scale of SI (from 0 to 9 points). When forming it, the scale of scores RL were taken into account as well as additional features of SI (per—1 point), the high probability of SI development is defined by the presence of ≥5 points according to SI scale Table 2 [8].

**Table 2:** Method of integral SI-scale calculation.

Phenomenon	Criterion	Points	Comments
SIR levels	RL- Scale 0 to 5 points	2–5	0–1 points exclude SI
Microthrombogenesis	D-dimers > 500 ng/ml (normal to 250 ng/ml)	1	Or presence of DIC-syndrome.
Distress-response	Cortisol >1380 or <100 nmol/l	1	Norm in blood plasma: 138–690 nmol/l.
Systemic alteration	Troponin I ≥ 0.2 ng/ml and/or myoglobin ≥ 800 ng/ml (normal to 25 ng/ml)	1	Troponin I is not included in myocardial infarction
Multiple organ dysfunction	Scale SOFA and/or other criteria MODS	1	Was defined by the clinicians

High probability of SI ≥5 points. For determining the plasma concentration of troponin I, myoglobin, cortisol, D-dimer the system «Immulıte» was used.

In general, the SI scale is an open system, where other criteria can be used, comparative by their pathogenetic and diagnostic importance. In particular, in sepsis, the expediency to use markers of cell stress, more specific for infections, such as procalcitonin and presepsin may appear [9, 10]. You can also vary the number of indicators (≥3) to determine the RL. This also applies to the SI- scale criteria in regards to their private indicator and scale replacement of individual units, e.g., distress-reaction of the neuroendocrine system’s shifting to a probably more specific component of SI process-complex. Different criteria of resuscitation syndrome (MODS—by definition) can be used. For example, integral features of DIC syndrome more reliably confirm the presence of microthrombogenesis phenomenon, as compared with only one indicator of the scale SI—D-dimer.

Details of choice for specific criteria in this case were selected for all indicators of determination (except MODS) by the closed system for immunochemiluminometric assay «Immulite» (Siemens Medical Solutions Diagnostics, USA), and because of the flexibility and degree of scrutiny of these indicators. In general, to determine the specific reference range or criteria, the results of mathematical processing as well as expert evaluation of our own results and available literature data had been used.

### Patients

Patients with a variety of acute inflammatory diseases, with signs of SIRS, and the availability of critical complications or significant risk factors for their occurrence were included into the prospective study. Material for study was picked up in a number of medical clinics of Yekaterinburg, where the cases of MODS and SIRS were diagnosed. Studies of blood plasma for identifying the scale of SI-criteria and calculation of integral indices were carried out in the Institute of immunology and physiology of UB RAS. Thus, clinical findings, including the presence of critical complications and outcome of a disease were independent, and they were provided for clinicians after completing the analysis of the SI- scale in the studied groups of patients. In total, 422 patients were studied (345 present in the intensive care wards) with various septic (n-207) and aseptic pathologies (n-215) and 62 patients in two control groups. 78 cases were lethal (28-days mortality). The research was approved by the Ethics Committee of Institute Immunology and Physiology UB RAS (Protocol No 1-SI-08-2012) and patients’ informed written consents were obtained.

#### Noninfectious pathologies

The following groups of patients were marked out:

1. Control group, practically healthy people—blood donors, n = 50, aged 18–55 years (Mean ± σ): 34.1 ± 10.4 years;
2. Women in the process of uncomplicated labor, n = 12, mean age– 28.7 ± 6.4 years;
3. Pre-eclampsia in the III trimester of pregnancy, without critical complications for mother and child, the presence of SIRS, n = 22, age—30.0 ± 6.5 years;
4. The same, as № 3, but in the process of childbirth, n = 15, age– 29.9 ± 7.9 years;
5. Acute obstetric hemorrhage > 1 lıtre, patients with severe complications of pregnancy and childbirth: antenatal fetal death, premature detachment of the placenta, eclampsia, uterine rupture; the absence of severe shock and MODS in all cases, examination at the 1st day of complications, n = 13, age– 26.8 ± 1.7 years;
6. The same, as №5, but with the severe shock development (grade 2–3) and MODS, not responding to intensive therapy during the 1st day, n = 13, age– 30.0 ± 1.8 years;
7. Acute multiple injuries, 1–2 days of hospitalization in the intensive care unit (ICU), without the development of MODS, n = 37, mean age– 40.4 ± 14.3 years;
8. The same, as №7, but with MODS development at the 1st-2d day from the time of hospitalization, n = 38, age– 35.0 ± 13.3 years;
9. Multiple injuries of 5–7 days of hospitalization in the ICU, without MODS, n = 45, age– 37.9 ± 13.0 years;
10. The same, as №9, but with the development of MODS at 5–8 days from the time of hospitalization, n = 15, age– 44.5 ± 19.4 years;
11. Heart valves replacement (open heart surgery), examination at 12–24 hours from the start of operation, the presence of MODS and other critical complications during their stay in the ICU were not observed, n = 17, age– 35.9 ± 10.6 years.

#### Patients with sepsis

The following groups were studied:

1. Deep shin phlegmon—III-IV level of soft tissue damage in military men, in all patients signs of SIRS and MODS were shown (average score on a scale of SOFA– 3.6 –from 2 to 5 points, with the maximum possible value of the scale—24 points). The dominant etiological factor was S. aureus. The study was conducted immediately after the surgical treatment of the inflammatory focus. Deaths and shock states in the postoperative period were not observed, treatment was carried out only in the surgical department, n = 40, age– 19.0 ± 0.9 years.
2. Sepsis (bacterial), 1–2 days of hospitalization. Initial diseases: severe pneumonia, peritonitis, obstetrical sepsis, some other reasons. Some patients had dysfunction of one system only (severe sepsis), but no signs of MODS, n = 31, age– 41.1 ± 18.0 years, and all patients in this and in other groups went through intensive therapy in the ICU.
3. Severe sepsis with MODS, n = 46, mean age– 50.1 ± 16.6 years, and 1–2 days from hospitalization in the ICU;
4. The same + septic shock (the presence of hypotension, not responding to vasopressors), n = 14, age– 54.9 ± 16.4 years;
5. Sepsis without MODS, screening at the 5–7 day of hospitalization in the ICU, n = 12, age– 40.2 ± 15.7 years;
6. The same, but with MODS, n = 13, mean age– 37.7 ± 15.4 years;
7. Tertiary peritonitis with MODS, and prolonged subacute septic process—more than 14 days from the date of hospitalization to the ICU, n = 34, age -51.5 ± 16.6 years;
8. The same + development of septic shock, n = 17, mean age– 50.2 ± 15.6 years.

### Statistical analysis

Statistical analyses were performed using SPSS for Windows 15.0 (SPSS Inc., Chicago, IL, USA) and MedCalc version 7.4.4.1 for Windows (MedCalc Software). Data are presented as frequencies with percentages for categorical variables. Comparisons between groups were performed using Chi-square (χ^2^) test for categorical variables. Areas under receiver operating characteristic curve (AUROC) were used to evaluate the ability of CR, SI scale and SOFA score to discriminate survivors from nonsurvivors and were pairwise compared. Statistical significance was defined as a *p* value <0.05.

## Results

### The results of SI—scale use in noninfectious pathologies

Table 3 represents the data of SI scale use in a number of noninfectious pathologies, as a percentage (%) of some criteria manifestations, and mortality cases.

**Table 3:** Frequency distribution of SI-scale criteria and lethal outcomes (in %) in groups with aseptic pathology.

Groups	RL						Tro	Myo	D-d	Cor	SI	LO
	0	1	2	3	4	5						
Blood donors	**100**^1^	**0**^1^	0	0	0	0	0	0	0	0	0	0
Uncomplicated childbirth	**50**^1^	**50**^1^	0	0	0	0	0	0	0	0	0	0
Preeclampsia, 3^rd^ trimester	**77.3**^2^	**22.7**^2^	0	0	0	0	0	0	9.1	0	0	0
The same during childbirth	**26,7**^2^	**60**^2^	13.3	0	0	0	0	0	33.3	0	0	0
Obstetric hemorrhage	7.7	7.7	46.1	30.8	7.7	**0**^3^	**0**^3^	0	92.3	**15.4**^3^	**7.7**^3^	**0**^3^
The same+shock and MODS	0	7.7	15.4	30.8	7.7	**38.4**^3^	**69.2**^3^	23.1	100	**53.8**^3^	**92.3**^3^	**53.8**^3^
Injury 1–2 days	0	10.8	**43.3**^4^	32.4	**10.8**^4^	2.7	32.4	16.2	72.2	**13.5**^4^	**37.8**^4^	**0**^4^
The same + MODS	0	2.6	**21.1**^4^	39.5	**34.2**^4^	2.6	52.6	34.2	65	**36.8**^4^	**78.9**^4^	**26.3**^4^
Injury 5–7 days	0	15.6	57.8	24.4	**2.2**^5^	0	**13.3**^5^	2.2	**21.1**^5^	**15.6**^5^	**8.9**^5^	**0**^5^
The same + MODS	0	6.7	33.3	26.7	**33.3**^5^	0	**60**^5^	6.7	**71.4**^5^	**53.3**^5^	**66.7**^5^	**60**^5^
Open-heart surgery	0	0	0	41.2	29.4	29.4	n\c	11.8	23.5	17.6	41.2	0

RL-reactivity level, «Tro»-the level of troponin I >0.2 ng/ml, «Myo» –myoglobin level>800 ng/ml, «D-d» –D-dimer level>500 ng/ml, «Cor» –the level of cortisol >1380 nmol/l or <100 nmol/l, SI—systemic inflammation (≥5 points of SI-scale), LO—lethal outcomes, n/c—not considered, as troponin I is specific to the myocardium. Bold typed data are valid differences on criterion Chi-square (χ^2^, p<0.05):

^1^- Blood donors / Uncomplicated childbirth;

^2^—Preeclampsia, 3^rd^ trimester / The same during childbirth;

^3^—Obstetric hemorrhage / The same + shock and MODS;

^4^—Injury 1–2 days / The same + MODS;

^5^—Injury 5–7 days / The same + MODS.

In these cases, the obvious manifestations of SI as a typical pathological process can only be considered in respect of ICU patients Table 3. In the group of patients with obstetric hemorrhage not complicated by shock and MODS, only 1 case from 13 can be directly traced to SI, whereas the group with the presence of these complications exhibits the opposite result. This group is characterized by the RL-range fluctuation from 1 (deliberately excluding the presence of SI)—to RL-5 (hyperergic phase of flogogenic impact). In some patients from this group the process is characterized by the hyperacute disease course (so called "breakthrough"), which is characterized by rapid change of proinflammatory activity phases during the day (rapid changes of RL from 5 to 2 points, depending on the time of collecting the material at the first day of examination). Furthermore, in this dynamic variant of SI the development of hemorrhagic shock (primary, initiating system injury) goes ahead of the realization of SI mechanisms, starting a few hours earlier, because these mechanisms require engagement of a large number of inducible genes. This can probably explain one case without confirmed SI—it was examined once, approximately after the moment of acute blood loss and the moment of hemorrhagic shock, triggering a possible further development of SI (Table 3).

In groups with acute trauma (1–2 days from the moment of hospitalization) cases with detected SI were mostly found in the group with the development of MODS, the result is valid on criterion Chi-square (χ^2^, p <0.05), but the contrast between two groups of patients with MODS / without MODS is not very prominent (78.9% and 37.8%, respectively). More manifested differences between these groups were marked at the 5–7 day process (implementation of the phenomenon of secondary systemic damage). This applies to a particular manifestation of differences in SIR (especially, the RL-4), as well as indicators of tissue destruction, distress reaction, and microthrombogenesis (all differences were significant on criterion Chi-square (χ^2^, p <0.05). In groups without MODS there is a high percentage of tissue destruction (especially from troponin I), which indirectly indicates the possibility of latent development of microcirculatory disorders and no obvious signs of MODS (Table 3).

Systemic inflammation is not a fatal process in the early stages of its development, especially before the advent of the phenomenon of secondary systemic damage. This is confirmed by the results of "open heart surgery"-group examination (the character of primary damage is under control). In this case, despite the high value of RL (the phase of phlogogenic stroke with RL-4-5 is dominating) and manifestations of integral signs of SI– 41.2% (≥5 points on the scale SI) critical complications won’t be noted (Table 3).

### The results of SI—scale use in groups with sepsis

Table 4 presents the usage of the SI scale in some variants of sepsis.

**Table 4:** Frequency distribution of SI-scale criteria and lethal outcomes (in %) in groups with sepsis.

Groups	RL						Tro	Myo	D-d	Cor	SI	LO
	0	1	2	3	4	5						
Phlegmon 1–2 days, MODS	0	27.5	55	17.5	0	0	2.5	5	25	0	10	0
Sepsis 1–2 days	0	0	22.6	61.3	16.1	0	25.8	3.2	51.6	**6.5**^1^	**29**^1^	**0**^1^
The same + MODS	0	4.3	10.9	41.3	30.4	13.1	32.6	6.5	58.7	**30.4**^1^	**73.9**^1^	**23.9**^1^
Sepsis 5–7 days	0	**33.2**^2^	**50**^2^	16.7	**0**^2^	0	25	0	41.7	0	**0**^2^	**0**^2^
The same + MODS	0	**0**^2^	**7.6**^2^	46.2	**46.2**^2^	0	46.2	7.7	92.3	15.4	**92.3**^2^	**30.8**^2^
Septic shock 1–2 days	0	0	**7.1**^3^	**14.3**^3^	**42.9**^3^	**35.7**^3^	50	7,1	85,7	57,1	100	78,6
Tertiary peritonitis + septic shock	0	0	**35.3**^3^	**58.8**^3^	**5.9**^3^	**0**^3^	64.7	11.8	88.2	35.3	100	94.1
Tertiary peritonitis + MODS	0	0	14.7	64.7	**17.7**^4^	2.9	32.4	2.9	85.3	11.8	85.3	29.4

RL-reactivity level, «Tro» - the level of troponin I >0.2 ng/ml, «Myo» –myoglobin level >800 ng/ml, «D-d» –D-dimer level >500 ng/ml, «Cor» –the level of cortisol >1380 nmol/l or <100 nmol/l, SI—systemic inflammation (≥5 points of SI-scale), LO—lethal outcomes. Bold typed data is valid different on criterion Chi-square (χ^2^, p<0.05):

^1^—Sepsis 1–2 days / The same + MODS;

^2^—Sepsis 5–7 days / The same+ MODS;

^3^—Septic shock 1–2 days / Tertiary peritonitis + septic shock;

^4^—Sepsis 5–7 days + MODS / Tertiary peritonitis + MODS.

All patients of the "shin phlegmon" group are formally diagnosed with severe sepsis and MODS, but clinically the condition of the patients was not considered as critical and they were treated in the surgical department, in all cases the outcome was recovery. We have registered signs of SI in only 10% of cases, with relatively mild manifestation of SIR (RL-1-3), and a slight manifestation of other criteria SI (Table 4). Most likely that there will be a significant difference in terms of probability of mortality in MODS in SIRS cases in the surgical department and ICU. However, this also applies to verifying the “sepsis” diagnosis by the presence of SIRS criteria.

In groups of ICU patients, basic laws are the following: 100% confirmation of SI in both variants of septic shock (acute and tertiary peritonitis), more pronounced manifestation of SI criteria in MODS with a high level of mortality, lower levels of RL expression in patients with tertiary peritonitis (prevalence of immunosuppressive variations of disease). The overall picture is quite comparable to the identification of patterns of acute trauma (Table 4).

Thus, the high probability of SI developing (according to the scale SI ≥5 points) is typical for groups with a high percentage of deaths Tables 3 and 4. There were 78 reported deaths and 73 patients of this category (93.6%) recorded SI signs. The RL scale or its derived CR scale, as well as the SI scale, can play an independent role in probabilistic forecast of adverse outcomes in acute sepsis and trauma Table 5. It should be noted that the main task of our developed scales is not only to be used for diagnosis and prognosis for MODS development and outcome, but in the first place also to define a whole pathological image of a nosology. Moreover, the scales can be applicable to monitoring the process stages and phases, and this allows specific therapy to be applied in turn.

**Table 5:** Performance of CR-scale, SI-scale and SOFA score in predicting 10-day mortality.

Group	Days	AUROC (CI) P value		
		CR	SI scale	SOFA
Acute sepsis	1–2 (n = 129)	**0.835 (0.760–0.895) p<0.001**	0.864 (0.792–0.918) p<0.001	**0.962 (0.913–0.988) p<0.001**
	5–7 (n = 25)	0.935 (0.834–1.035) p = 0.007	0.935 (0.835–1.034) p = 0.007	0.946 (0.776–0.9930 p = 0.005
Injury	1–2 (n = 58)	0.853 (0.734–0.930) p = 0.001	0.780 (0.652–0.878) p = 0.006	0.850 (0.732–0.930) p = 0.001
	5–7 (n = 54)	0.945 (0.847–0.988) p<0.001	0.989 (0.914 to 0.995) p<0.001	0.964 (0.873 to 0.995) p<0.001

AUROC—area under the receiver operating characteristic curve; CI—confidence interval; bold typed data is significant difference by pairwise comparison.

## Discussion

From the standpoint of common pathology, as we believe, it is proper to consider SI from the perspective of a typical pathological process, which forms the theoretical foundation for the various syndromes’ models in critical care medicine. The most obvious manifestation of SI was observed during development as a "breakthrough"–variation. However, more typical development for SI is "punchıng," with gradual shifting of classical inflammation to the systemic inflammation.

SIRS criteria are less specific to the development of complications. Resuscitation syndromes criteria, including MODS, solve this problem somehow, but do not give a complete picture of SI pathogenesis, which hinders their structuring into a single system. One way to solve this problem is to consider the SI from the position of a typical pathological process, which differs from inflammation in the classic interpretation by the number of fundamental features.

A key element of SI pathogenesis is the phenomenon of "systemic inflammatory microcirculation," which eventually leads to the development of critical complications including multiple organ dysfunctions. Systemic inflammation is a major cause of death in the ICU, but it is not fatal, and the process can be stopped at the initial stages of its development by the methods of intensive care, and in some cases with a timely elimination of the primary factors (initiation) of systemic damage. Obvious clinical manifestation of SI is heavy shock. However, critical for life microcirculatory disorders can be developed under normal macrohemodynamics parameters [11]. This makes it impossible to bind SI to a particular clinical picture of the disease. More difficult is to describe in detail is the pathogenesis of compound SI-process-complex, but it is possible to designate its principal pathogenetic image and try to recognize this process in specific clinical situations. In this regard, the scale of SI (≥5 points), basing on the definition of indirect signs together, identifies cases with a high probability of SI.

There were 422 patients examined with different septic and aseptic pathologies, with the presence of SIRS criteria, and with risk factors or with manifestations of critical complications already present. In 190 cases the signs of SI were detected, the average mortality rate in detecting SI was 38.4% (n-73). Only 5 cases of death were not confirmed by the presence of SI. In 100% of cases, SI signs were noticed together with developing septic shock (n-31; of them fatal -27).

In at least several patients in the group of "massive obstetric hemorrhage with shock," there was hyper acute SI developing according to the «breakthrough» variant, with a rapid change of activity phases—from the RL-5 to the minimum for SI—RL-2, and a high level of negative outcomes. However, as already noted, in most cases, the transition from being mostly protective to the organism’s system part of classical inflammation to the obvious manifestations of SI does not happen discretely (version of "punching"). A special place in this variant of SI takes tertiary peritonitis (long and subacute sepsis) which is characterized by (especially in septic shock) the high percentage of SI manifestations and the high level of deaths on the background prevalence of the depressive phase SI (RL-2-3).

Hyperergic SI variants are more typical in acute sepsis with high meanings of RL, especially during the septic shock development (RL-5–35.7%), as well as in an undefined state with fuzzy manifestations of SI-symptoms in many cases of acute sepsis. This state of the body can be defined as pre-SI, which is a risk factor for the development of more obvious manifestations of SI. To fix the pre-SI, the SI- scale can be used also, allocating in it a transition zone (3–4 points). To specify this zone we need to monitor the process and raise additional data. In this case, the SI-scale (≥3 points) will cover the overwhelming majority of resuscitation pathologies that will require differentiating signs of pre-SI from a number of pathologies that are not directly related to the development of acute SI, including some chronic diseases [6]. As noted above, the SI-scale is open and flexible, and, using different applied integrated criteria, it can be adapted to computer programs for information support in clinical decision making.

The creation of abstract criteria that can integrally characterize complex types of pathogenetic processes is likely one of the key tasks in general pathology. Another more fundamental problem, in our opinion, is a description of the common pathological process, as theoretical and methodological basis of various pathologies, and its differentiation from clinical superstructure: different syndromal and nosological models. This is particularly important in dealing with both theoretical and practical problems of systemic inflammation.

In this paper, we have not discussed the problem of chronic systemic inflammation, as this topic requires a separate study [6].

The work was supported by Act 211 Government of the Russian Federation, contract No 02.A03.21.0006.
